# Association between Nutrition and Health Knowledge and Multiple Chronic Diseases: A Large Cross-Sectional Study in Wuhan, China

**DOI:** 10.3390/nu15092096

**Published:** 2023-04-27

**Authors:** Shanshan Wang, Yating Wu, Mengdie Shi, Zhenyu He, Liping Hao, Xiaomin Wu

**Affiliations:** 1 Hubei Key Laboratory of Food Nutrition and Safety, MOE Key Laboratory of Environment and Health, Department of Nutrition and Food Hygiene, School of Public Health, Tongji Medical College, Huazhong University of Science and Technology, Wuhan 430030, China; doubleshan@hust.edu.cn (S.W.); haolp@mails.tjmu.edu.cn (L.H.); 2 Wuhan Center for Disease Prevention and Control, Institute of Environmental Health and Food Safety, Wuhan 430022, China; cherrywu521@gmail.com (Y.W.); mengdie.shi@whcdc.org (M.S.); food@whcdc.org (Z.H.)

**Keywords:** nutrition and health knowledge, chronic diseases, health literacy, diabetes, hypertension, dyslipidemia

## Abstract

Nutrition and health knowledge (NHK) is linked to people’s dietary behavior and health outcomes. However, studies on the associations between NHK and chronic diseases are limited. This study aimed to examine the association of NHK with five specific chronic diseases (diabetes/hyperglycemia, hypertension, dyslipidemia, coronary heart disease (CHD), and stroke) in central China. Individual NHK and disease status were investigated using a self-reporting questionnaire. We further added up the number of chronic diseases and used this as a secondary outcome. A total of 21,559 adults were enrolled in this cross-sectional study. NHK score was significantly inversely associated with diabetes/hyperglycemia, hypertension, CHD, and stroke (all *p*-trends < 0.001). Moreover, an inverse association was found between NHK and the number of chronic diseases, especially among responders with three or more chronic diseases. Stratified analyses showed that the above association was more likely to be stronger among younger, female, highly educated, and inner-city residents. However, NHK was negatively associated with dyslipidemia in less educated people and positively correlated with dyslipidemia in highly educated people. NHK showed an inverse relationship with specific chronic diseases and the number of chronic diseases. Improving NHK might be a key strategy for easing the global burden of chronic diseases.

## 1. Introduction

It is estimated that about 41 million people die from non-communicable and chronic diseases, equivalent to 74% of all-cause deaths globally [1]. Cardiovascular and cerebrovascular diseases, diabetes, cancers, and chronic respiratory diseases are the major non-communicable and chronic diseases that have brought an ever-mounting burden to China [2]. Although China has made good progress in improving population health, the dramatic shift in Chinese dietary structure in recent decades makes it a challenge to reduce the prevalence and incidence of chronic diseases [3,4]. Worldwide, approximately 12.2% of total disability-adjusted life years (DALYs) in men and 9.0% of total DALYs in women are attributed to poor diet [5]. Given the substantial public health costs of nutrition-related chronic diseases (NRCDs), improving people’s dietary habits is urgently needed.

According to social cognitive theory [6], to implement a specific behavior, a person must know what the behavior is and how to engage in it. In terms of diet, based on this theory, a person must be aware of what a favorable diet is before being asked to follow it accurately. Therefore, an increasing number of studies have focused on the potential relationship between dietary quality and modifiable cultural factors, such as nutrition literacy, which are deemed to be key factors in explaining differences in dietary behavior [7,8]. Cutler et al. [9] used six datasets from the US and the UK to examine the possible explanations for the association between education and health behaviors and found that knowledge and measures of cognitive ability could explain up to 30% of the educational gradient in health behaviors, whereas the impact of nutrition education on dietary practices was equivocal. Data from the 2013 China Chronic Disease Surveillance Survey suggested that lower health literacy and irregular breakfast and lunch habits were associated with lower vegetable and fruit consumption [10]. Similarly, several observational studies have shown a positive and significant association between nutrition and health knowledge (NHK) and overall dietary habits [11,12,13], while others reported no significant correlation between NHK and food choices [14]. Although NHK may be a necessary but insufficient factor for changing behavior, it is considered to be a pivotal mediator between nutrition and health literacy and health outcomes.

It is well accepted that NHK, along with beliefs and attitudes, plays a central role in health-related behaviors and health outcomes. However, current studies on the relationship between NHK and NRCDs are sparse and controversial [4,15]. A Chinese cross-sectional study found a negative relationship between nutrition literacy and multimorbidity [16]. An observational study including 500 patients in Požega, Croatia, observed that health literacy was inversely associated with hypertension and type 2 diabetes mellitus (T2DM), but not with obesity [17]. Some studies have explored the association between NHK and cholesterol levels, with conflicting results [18,19]. However, few studies have examined the association between NHK and cardiovascular and cerebrovascular diseases, particularly with regard to nutritional aspects. Given the key role of NHK in the prevention and management of NRCDs, it is of great significance to explore the association of NHK with NRCDs to seek effective measures to improve people’s dietary behaviors and promote health.

Our previous research has evaluated the awareness level of NHK and its influencing factors among Wuhan residents [20], whereas, to the best of our knowledge, few studies have examined the association between NHK and multiple NRCDs in a large population-based study. The current study aimed to examine the relationship between NHK and the prevalence of diabetes/hyperglycemia, hypertension, dyslipidemia, coronary heart disease (CHD), and stroke, as well as the number of chronic diseases among adults in Wuhan, China.

## 2. Materials and Methods

### 2.1. Study Design and Participants

The cross-sectional study of the China Nutrition and Health Knowledge Survey (CNHKS) was conducted in April 2021 and intended to investigate the NHK level and its influencing factors among Chinese people. Permanent residents aged 18–64 years from Wuhan, a representative first-tier city in central China, were analyzed in this study. According to the results from the sixth national population census in China, the sample selection conformed to the proportion of half male and half female, and the proportion of 18–24-, 25–34-, 45–54-, 35–44-, and 55–64-year-olds was 20%, 20%, 20% 30%, and 10%, respectively. Participants came from 15 monitoring sites in Wuhan and were representative in terms of age, gender, and regional distribution. The details of sample selection can be found elsewhere [20]. Responders who agreed to participate in the survey and were clearly aware of their health status were enrolled in the study (n = 21,563). After excluding those with extreme NHK scores (score < fifth percentile, or score > ninety-fifth percentile, n = 4), finally, 21,559 participants were included in the analysis. All participants were informed about the study, and consent was obtained before the survey. This study was approved by the Ethics Review Committee of the Wuhan Centers for Disease Prevention and Control (approval number: WHCDCIRB-K-2021033).

### 2.2. Questionnaire and Scoring

NHK scores were measured based on the “Nutrition and health knowledge questionnaire” designed by the National Institute for Nutrition and Health, Chinese Center for Disease Control and Prevention. This structured questionnaire included questions concerning respondents’ sociodemographic characteristics (i.e., age, gender, educational level), disease status (including diabetes/hyperglycemia, hypertension, dyslipidemia, CHD, and stroke), NHK, and way of obtaining knowledge. The 32-item survey of NHK includes questions involving the following four parts: (a) dietary guideline recommendations (items 1–18); (b) food and nutrients (items 19–22); (c) nutrition and disease prevention (items 23–26); and (d) nutrition skills (items 27–32). The specific items of the NHK part are shown in Table S1. Each item was scored according to the weight of the expert consultation, and the final score was calculated. The scoring details can be seen in our previous study [20]. The total NHK scores range from 0 to 100, with a score of 75 or above being defined as “awareness”, while other scores are defined as “unawareness”.

### 2.3. Outcome Variable

Chronic diseases related to suboptimal nutrition were selected as health outcomes, which were assessed through self-reporting. Respondents were asked whether they had ever been diagnosed with any of the five selected chronic diseases, or two or more. The chronic diseases examined in the present study were diabetes/hyperglycemia, hypertension, dyslipidemia, CHD, and stroke. To better understand the relationship between NHK and chronic diseases, we further added up the number of chronic diseases reported by each responder and used this as a secondary outcome, including “0”, “1”, “2”, and “3 or more” of the five chronic diseases.

### 2.4. Covariates

Information on covariates was obtained at enrolment, including age, gender, education level, occupation, residence, obtaining knowledge from an app, and participating in educational activities. Age was treated as a continuous variable. Gender (male and female), education level (junior school diploma or below, high school diploma, junior college diploma, and bachelor’s degree or above), occupation (medical workers, catering service workers, other health-related workers, educational workers, and others), and residence (downtown area and remote area) were treated as categorical variables. The variables of knowledge acquisition from an app and educational activities were defined according to the question “Do you often get nutrition and health knowledge from an app?” and “Do you often participate in education activities?”, and treated as dichotomous variables (Yes/No).

### 2.5. Quality Control

After the review of the expert group, pre-survey, and reliability and validity test, the constructed questionnaire was developed. Before investigation, all of the investigators had to participate in professional training and evaluation, and the subjects were informed of the purpose and completion method before answering the questionnaire. After completion of a questionnaire, an on-site inspection was implemented rapidly. When all monitoring sites had completed the survey, the quality control personnel of the Wuhan Centers for Disease Prevention and Control carried out a call-back interview with the respondents in line with a specifically designed review questionnaire. If three or more of the return visit questionnaires in a community did not meet the criteria, the community survey was considered substandard and needed to be reinvestigated.

### 2.6. Statistical Analysis

Categorical data were expressed as counts and percentages, and compared using the chi-squared test. Continuous data with normal distribution are represented as mean ± standard deviation (SD), and were compared by using one-way analysis of variance, while continuous data with skewed distribution were described as median (interquartile range, IQR), and the Mann–Whitney U test was used for comparison between groups.

The NHK score was assessed as both categorical (tertiles of NHK, with the lowest tertile as the reference group, dichotomy of NHK, cut-off point of 75) and continuous variable (per SD increase). Binary logistic regression was used to examine the association between NHK score and specific chronic diseases. Multinomial logistic regression was used to analyze the relationship between NHK score and the number of chronic diseases, with the non-chronic disease group as the reference. The results of regression analysis were expressed as odds ratios (ORs) and 95% confidence intervals (95% CIs). Multivariate models were conducted as follows: (a) crude model; (b) adjusted model for adjustments of responder’s age, gender, education level, occupation, residence, obtaining knowledge from an app, and participating in educational activities. Furthermore, to test the significance of linear trends across tertiles, the median value of each tertile of NHK scores was considered to be a continuous variable.

To evaluate the potential modification effect, stratified analyses were conducted by the median value of age (<39 and ≥39 years), sex, education level (high school diploma or below and junior college diploma or above), and residence (downtown area and remote area). The likelihood ratio tests were used to assess the interactions between stratified variables and NHK score. All statistical analyses were performed using SPSS version 25.0 (IBM Corporation, Armonk, NY, USA) and R software, version 4.2.2. A two-tailed value of *p* < 0.05 was considered indicative of statistical significance.

## 3. Results

### 3.1. Characteristics of Participants

Table 1 summarizes the characteristics of the study sample by tertiles of NHK scores. The median NHK score for the whole sample was 67.0. Compared with the lowest tertile of NHK scores (<51.5), those with the highest tertile of NHK scores (≥77.0) were more likely to be female, more educated, having a higher proportion of educators and people living downtown, acquiring knowledge from an app, and participating in educational activities. The prevalence of diabetes/hyperglycemia, hypertension, dyslipidemia, CHD, and stroke in this study was 4.9%, 14.5%, 7.6%, 1.6%, and 0.7%, respectively. Compared with the lowest tertile of NHK scores, those with the higher scores had a lower proportion of diabetes/hyperglycemia, hypertension, CHD, and stroke and a higher proportion of dyslipidemia. Moreover, responders with higher NHK scores reported fewer chronic diseases than those with lower NHK scores (Table 1).

### 3.2. Association of NHK with Five Specific Chronic Diseases 

Table 2 demonstrates that NHK scores were significantly negatively associated with diabetes/hyperglycemia, hypertension, CHD, and stroke in both the crude and adjusted models. After adjusted potential confounders, compared to the lowest tertile, the adjusted ORs (95% CIs) of diabetes/hyperglycemia, hypertension, CHD, and stroke in the highest tertile were 0.50 (0.42, 0.59), 0.56 (0.50, 0.62), 0.35 (0.26, 0.47), and 0.24 (0.14, 0.41), respectively (all *p*-trends < 0.001). Similarly, per SD increase in NHK score was associated with diabetes/hyperglycemia, hypertension, CHD, and stroke, with adjusted ORs (95% CIs) of 0.70 (0.66, 0.74), 0.77 (0.74, 0.80), 0.56 (0.51, 0.62), and 0.50 (0.43, 0.58), respectively. However, compared to the lowest tertile, the OR (95% CI) of dyslipidemia in the highest tertile was 1.18 (1.05, 1.34) in the crude model (*p*-trends = 0.007), and such significant associations disappeared in the adjusted model (Table 2).

According to the cut-off of 75 points, responders were divided into an unawareness group (score < 75) and an awareness group (score ≥ 75). A total of 4987 (23.1%) responders were aware of NHK in this study (Table 3). Further exploration of the association between NHK and the five chronic diseases under this grouping is shown in Table 3. Similarly, we found a significant inverse association between NHK and diabetes/hyperglycemia, hypertension, CHD, and stroke under this grouping (all *p*-trends < 0.001). A significant positive association between NHK and dyslipidemia was observed in the crude model (*p*-trends = 0.002), but not in the adjusted model (*p*-trends = 0.283).

### 3.3. Association of NHK with the Number of Chronic Diseases

Compared with the lowest tertile of NHK scores, those with the highest scores had a lower proportion of any one (15.6% vs. 24.6%), two (2.7% vs. 4.3%), or three or more (0.7% vs. 1.2%) chronic diseases (Table 1). Multinomial logistic regression for the association between NHK and the number of chronic diseases is presented in Table 4. With the group without chronic diseases as a reference, compared to the lowest tertile of NHK score, the highest tertile was negatively associated with the prevalence of any one, two, or three or more chronic diseases, especially among responders with three or more chronic diseases (OR = 0.59, 95% CI = 0.54–0.65; OR = 0.54, 95% CI = 0.44–0.64; OR = 0.45, 95% CI = 0.31–0.65, respectively). When NHK score was analyzed as a continuous variable, compared with those without chronic diseases, the adjusted ORs (95% CIs) of subjects with any one, two, or three or more chronic disease were 0.71 (0.69, 0.74), 0.73 (0.67, 0.78), and 0.70 (0.61, 0.80), respectively.

### 3.4. Stratified Analyses

**Figure 1:**
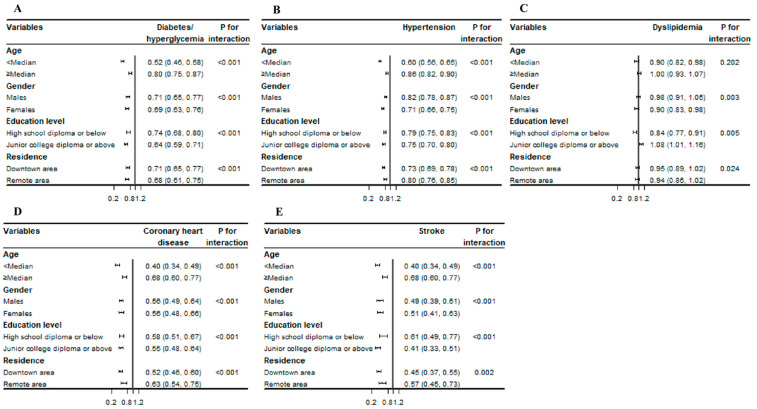
Stratified analysis of the association between per SD increment in NHK and the prevalence of chronic diseases. (**A**) Diabetes/hyperglycemia; (**B**) hypertension; (**C**) dyslipidemia; (**D**) coronary heart disease; and (**E**) stroke. Adjusted for age, gender, education level, occupation, residence, knowledge acquisition from an app, and educational activities, and likelihood ratio tests for the interactions between stratified variables and NHK score.

To assess whether other confounding factors modified the association between NHK and chronic diseases, we conducted stratified analyses by age, gender, education level, and residence. The results showed that the correlation between NHK and five chronic diseases was significantly modified by age, gender, education level, and residence (except for no interactions of NHK with age in dyslipidemia). The inverse association between NHK and chronic diseases was also detected in the subgroup analyses, and it was stronger in younger, female (except for stroke), less educated (except for dyslipidemia), and urban residents (except for diabetes/hyperglycemia). Moreover, we found that NHK was negatively correlated with dyslipidemia in subjects with a high school diploma or below (OR = 0.84, 95% CI = 0.77–0.91) and positively correlated with dyslipidemia in subjects with a junior college diploma or above (OR = 1.08, 95% CI = 1.01–1.16; *p* for interaction <0.001) (Figure 1).

The association between NHK score and the number of chronic diseases in the subgroup was essentially consistent with the whole study population, but there were no significant associations between NHK score and the odds ratios of three or more diseases in subjects who were older and lived in a remote area (Figure S1).

## 4. Discussion

In this large cross-sectional study, NHK was inversely associated with diabetes/hyperglycemia, hypertension, CHD, and stroke after adjusting for potential confounding factors. These results remained stable after stratifying by age, gender, education level, and residence, while NHK was negatively correlated with dyslipidemia in subjects with lower education levels and positively correlated with dyslipidemia in subjects with higher education levels. Moreover, we found that a lower NHK score was associated with a higher number of chronic diseases.

It is widely accepted that individuals with lower NHK tend to report poorer health than those with adequate NHK [21,22,23]. According to our findings, we found responders with higher NHK scores were more likely to report a lower prevalence of diabetes/hyperglycemia, hypertension, CHD, and stroke, which was consistent with Lovrić’s finding that patients with T2DM or hypertension had less health literacy than those without T2DM or hypertension [17]. Similarly, Patel et al. [24] found that elderly patients with a lower score for nutrition literacy tended to have a higher proportion of hypertension. An observational study of 82 50-year-olds showed that higher education and nutrition literacy were inversely associated with the risk of cardiovascular disease [25]. However, a previous study by Itzkovitz et al. found that people with type 1 diabetes reported greater food literacy than controls [26]. Additionally, Chen et al. observed that adults with diet-related chronic diseases reported higher diet quality than nonpatients, especially those with better nutrition knowledge and beliefs [8]. These inconclusive findings might be partially explained by heterogeneity in study settings, population characteristics, research methodologies, and scales used to measure NHK. Moreover, it is important to note that, to date, few epidemiological studies have evaluated the relationship between NHK and chronic disease in the whole population, and most studies have been limited to specific groups such as elderly patients [24,27] or students [22,28], making it difficult to generalize the results to other age groups.

Several works in the literature have addressed the association between nutrition and health literacy and multiple chronic diseases, with controversial results. In this study, our results suggest an inverse relationship between NHK and the reported number of chronic diseases, which is consistent with the finding from a Chinese cross-sectional study of 1490 elderly people that nutrition literacy was significantly negatively related to the prevalence of multimorbidity [16], whereas Hudon et al. observed no association between health literacy and multimorbidity when adjusting for age and family income [29]. An observational survey of 2923 older adults reported that inadequate health literacy was associated with diabetes and heart failure, but not with hypertension, arthritis, or lung disease [28]. To summarize, there is a possibility that NHK and even nutrition and health literacy are related to multimorbidity. However, to the best of our knowledge, few studies have examined the association between nutrition and health literacy and chronic diseases, particularly with regard to nutrition knowledge.

It should be clearly recognized that the measurements of NHK are complex and heterogeneous, and influenced by a variety of factors such as sociodemographic characteristics and cultural background. In the stratified analysis, we found that people who were young, female, highly educated, and living downtown tended to have more pronounced associations. Our previous study demonstrated that people aged 25–44 who were female, more educated, and living downtown had higher NHK scores, which could partly explain these findings [20]. However, the present findings are in line with some studies [17,30,31] but not others [32,33]. Intriguingly, we found that NHK was positively correlated with dyslipidemia in subjects with higher education levels, which aligned with the findings of Li et al. [34]. This consequence may be related to the higher economic level accompanied by excess nutrition among people with higher education levels. Furthermore, studies have shown that people with more education spend more time sitting and are more likely to report cardiovascular disease, which could be another explanation for these findings [35]. Nevertheless, our finding of NHK related to dyslipidemia should be interpreted with caution as it reflects a subgroup finding, and no association was observed in the adjusted model. Additional studies, especially randomized controlled trials, are needed to confirm our conclusion.

Suboptimal NHK makes it a challenge to improve people’s dietary habits and further promote their health. Health experts have been committed to examining the validity of health promotion approaches for improving people’s behavior and preventing NRCDs for a long time. However, the prevalence of most NRCDs, including diabetes and cardiac–cerebral vascular disease, continues to rise in China. Limited to the gap between research and practice, other effective nutrition and health strategies are urgently needed [36]. A systematic review by Mackey et al. [37] reported that in the initial stages of self-management, health-related knowledge, self-efficacy, and belief are three indispensable factors and crucial mediators between health literacy and health outcomes [38]. Many experts also point out that awareness of dietary guidelines, food choices, and nutritional skills can help people implement healthy dietary patterns [39,40]. Collectively, as improving NHK level may help reduce the prevalence of NRCDs by improving people’s nutrition awareness and dietary behaviors, it would be of great significance for policymakers, researchers, and other healthcare workers to develop effective NHK assessment tools. Meanwhile, our evidence emphasizes the significance of applying NHK to practice according to the needs and possibilities of each individual, as well as their NHK level and sociodemographic characteristics.

This study has several strengths, which include the large sample size and representative sample, validated questionnaires, and rigorous approaches for quality control. However, our study also has certain limitations. First, due to the cross-sectional design, we could not suggest a causal association between NHK and chronic diseases. It cannot be excluded that the presence of chronic diseases such as diabetes as a health outcome may have the opposite causal effect to improved NHK through regular healthcare visits and health guidance. Second, although quality control was strictly executed throughout the survey, some information biases were inevitably generated by the self-administered questionnaire. Third, the definitions and measurements of NHK and nutrition literacy were determined using different scales. Therefore, the results of this study need to be treated with caution and verified in more prospective studies. Finally, although we adjusted some common covariables in the multivariable analysis as much as possible, residual confoundings such as body mass index remained. 

## 5. Conclusions

In conclusion, this large cross-sectional study demonstrated that NHK was inversely associated with diabetes/hyperglycemia, hypertension, CHD, and stroke, as well as the number of chronic diseases in the whole population, while NHK was negatively correlated with dyslipidemia in less educated people and positively correlated with dyslipidemia in highly educated people. Our findings support the proposal of improving people’s NHK as a possible tool for health promotion, independent of other entrenched risk factors such as socioeconomic characteristics.

## Figures and Tables

**Table 1:** Characteristics of the sample according to tertiles of NHK ^a^.

Variables	Overall	Tertiles of the Level of Nutrition and Health Knowledge			*p* Value ^b^
		T1 (n = 7414)	T2 (n = 7058)	T3 (n = 7087)	
NHK score	67.0 (57.0, 74.0)	51.5 (44.0, 57.0)	67.0 (64.5, 69.5)	77.0 (74.0, 80.5)	<0.001
Age (years)	39.5 ± 12.3	39.9 ± 12.8	39.3 ± 12.3	39.1 ± 11.8	<0.001
Gender					<0.001
Male	10,219 (47.4)	3922 (52.9)	3303 (46.8)	2994 (42.2)	
Female	11,340 (52.6)	3492 (47.1)	3755 (53.2)	4093 (57.8)	
Education level					<0.001
Junior school diploma or below	4127 (19.1)	1977 (26.7)	1262 (17.9)	888 (12.5)	
High school diploma	5249 (24.3)	2055 (27.7)	1780 (25.2)	1414 (20.0)	
Junior college diploma	4927 (22.9)	1646 (22.2)	1648 (23.3)	1633 (23.0)	
Bachelor’s degree or above	7256 (33.7)	1736 (23.4)	2368 (33.6)	3152 (44.5)	
Occupation					<0.001
Medical workers	2754 (12.8)	1066 (14.4)	742 (10.5)	946 (13.3)	
Catering service workers	907 (4.2)	429 (5.8)	290 (4.1)	188 (2.7)	
Other health-related workers	452 (2.1)	232 (3.1)	114 (1.6)	106 (1.5)	
Educational workers	1800 (8.3)	461 (6.2)	627 (8.9)	712 (10.0)	
Others	15,646 (72.6)	5226 (70.5)	5285 (74.9)	5135 (72.5)	
Residence					<0.001
Downtown area	10,912 (50.6)	3392 (45.8)	3532 (50.0)	3988 (56.3)	
Remote area	10,647 (49.4)	4022 (54.2)	3526 (50.0)	3099 (43.7)	
Knowledge acquisition from an app	17,308 (80.3)	5363 (72.3)	5901 (83.6)	6044 (85.3)	<0.001
Educational activities	4825 (22.4)	1302 (17.6)	1491 (21.1)	2032 (28.7)	<0.001
Diabetes/hyperglycemia	1064 (4.9)	517 (7.0)	315 (4.5)	232 (3.3)	<0.001
Hypertension	3123 (14.5)	1421 (19.2)	939 (13.3)	763 (10.8)	<0.001
Dyslipidemia	1648 (7.6)	522 (7.0)	543 (7.7)	583 (8.2)	0.027
Coronary heart disease	354 (1.6)	210 (2.8)	81 (1.1)	63 (0.9)	<0.001
Stroke	143 (0.7)	93.0 (1.3)	33.0 (0.5)	17.0 (0.2)	<0.001
0 of 5 chronic diseases	16,507 (76.0)	5205 (70.2)	5561 (78.8)	5741 (81.0)	<0.001
1 of 5 chronic diseases	4103 (19.0)	1802 (24.3)	1197 (17.0)	1104 (15.6)	<0.001
2 of 5 chronic diseases	742 (3.4)	320 (4.3)	229 (3.2)	193 (2.7)	<0.001
3 or more of 5 chronic diseases	207 (1.0)	87 (1.2)	71 (1.0)	49 (0.7)	0.011

^a^ Values are expressed as mean ± SD, n (%), or median (IQR). ^b^ Values were obtained by using the chi-square test for categorical variables, ANOVA test for continuous variables with normal distribution, and Mann–Whitney U test for continuous variables with skewed distribution. T, tertile; NHK, nutrition and health knowledge.

**Table 2:** Association between NHK and specific chronic diseases ^a^.

Model	Diabetes/Hyperglycemia	Hypertension	Dyslipidemia	Coronary Heart Disease	Stroke
Crude model					
T1	1.00	1.00	1.00	1.00	1.00
T2	0.62 (0.54, 0.72)	0.65 (0.59, 0.71)	1.10 (0.97, 1.25)	0.40 (0.31, 0.52)	0.37 (0.25, 0.55)
T3	0.45 (0.39, 0.53)	0.51 (0.46, 0.56)	1.18 (1.05, 1.34)	0.31 (0.23, 0.41)	0.19 (0.11, 0.32)
*p*-trend ^b^	<0.001	<0.001	0.007	<0.001	<0.001
Per SD increase	0.67 (0.64, 0.71)	0.75 (0.72, 0.77)	1.01 (0.96, 1.06)	0.54 (0.49, 0.59)	0.44 (0.38, 0.51)
*p* value	<0.001	<0.001	0.841	<0.001	<0.001
Adjusted model ^c^					
T1	1.00	1.00	1.00	1.00	1.00
T2	0.69 (0.59, 0.80)	0.70 (0.63, 0.77)	1.05 (0.92, 1.20)	0.45 (0.35, 0.59)	0.45 (0.30, 0.68)
T3	0.50 (0.42, 0.59)	0.56 (0.50, 0.62)	1.03 (0.90, 1.17)	0.35 (0.26, 0.47)	0.24 (0.14, 0.41)
*p*-trend	<0.001	<0.001	0.667	<0.001	<0.001
Per SD increase	0.70 (0.66, 0.74)	0.77 (0.74, 0.80)	0.96 (0.91, 1.01)	0.56 (0.51, 0.62)	0.50 (0.43, 0.58)
*p* value	<0.001	<0.001	0.099	<0.001	<0.001

^a^ Values are represented as ORs (95% CIs) from logistic regression models. ^b^ Tests for linear trends were conducted by creating a continuous variable using the median value of each tertile. ^c^ Adjustments for age, gender, education level, occupation, residence, knowledge acquisition from an app, and educational activities. T, tertile; NHK, nutrition and health knowledge; SD, standard deviation.

**Table 3:** Association between nutrition and health awareness (score < 75 or ≥75) and specific chronic diseases ^a^.

Model	Total	Diabetes/ Hyperglycemia	Hypertension	Dyslipidemia	Coronary Heart Disease	Stroke
Crude model						
Unawareness	16,572 (76.9)	1.00	1.00	1.00	1.00	1.00
Awareness	4987 (23.1)	0.51 (0.43, 0.61)	0.60 (0.55, 0.67)	1.20 (1.07, 1.35)	0.46 (0.33, 0.63)	0.33 (0.19, 0.59)
*p*-trend ^b^		<0.001	<0.001	0.002	<0.001	<0.001
Adjusted model ^c^						
Unawareness	16,572 (76.9)	1.00	1.00	1.00	1.00	1.00
Awareness	4987 (23.1)	0.56 (0.46, 0.67)	0.65 (0.58, 0.72)	1.07 (0.95, 1.21)	0.52 (0.37, 0.72)	0.41 (0.23, 0.73)
*p*-trend		<0.001	<0.001	0.283	<0.001	0.003

^a^ A total score of 75 or above is defined as “awareness”, with other scores defined as “unawareness”. Values are represented as ORs (95% CIs) from logistic regression models. ^b^ Tests for linear trends were conducted by creating a continuous variable using the median value of each tertile. ^c^ Adjustments for age, gender, education level, occupation, residence, knowledge acquisition from an app, and educational activities.

**Table 4:** Association between NHK and the number of chronic diseases through multinomial logistic regression ^a^.

Model	Number of Chronic Diseases		
	1	2	≥3
Crude model			
T1	1.00	1.00	1.00
T2	0.62 (0.57, 0.68)	0.67 (0.56, 0.80)	0.76 (0.56, 1.05)
T3	0.56 (0.51, 0.60)	0.55 (0.46, 0.66)	0.51 (0.36, 0.73)
*p*-trend ^b^	<0.001	<0.001	<0.001
Per SD increase	0.71 (0.68, 0.73)	0.75 (0.70, 0.80)	0.74 (0.65, 0.84)
*p* value	<0.001	<0.001	<0.001
Adjusted model ^c^			
T1	1.00	1.00	1.00
T2	0.66 (0.61, 0.73)	0.68 (0.56, 0.81)	0.76 (0.55, 1.05)
T3	0.59 (0.54, 0.65)	0.54 (0.44, 0.65)	0.45 (0.31, 0.65)
*p*-trend ^b^	<0.001	<0.001	<0.001
Per SD increase	0.71 (0.69, 0.74)	0.73 (0.67, 0.78)	0.70 (0.61, 0.80)
*p* value	<0.001	<0.001	<0.001

^a^ Values are represented as ORs (95% CIs) from multinomial logistic regression, and the group without chronic disease are regarded as reference. ^b^ Tests for linear trends were conducted by creating a continuous variable using the median value of each tertile. ^c^ Adjustments for age, gender, education level, occupation, residence, knowledge acquisition from an app, and educational activities. NHK, nutrition and health knowledge; T, tertile; SD, standard deviation.

## Data Availability

The data that support the findings of this study are available from the corresponding author upon reasonable request.

## Supplementary Materials

The following supporting information can be downloaded at: https://www.mdpi.com/article/10.3390/nu15092096/s1. Table S1: Items of nutrition and health knowledge; Figure S1: Stratified analysis of the association between per SD increment in NHK and the number of chronic diseases.
