# Supplementary material for: Association between Nutrition and Health Knowledge and Multiple Chronic Diseases: A Large Cross-Sectional Study in Wuhan, China
Source: Nutrients. 2023 Apr 27;15(9):2096. doi: 10.3390/nu15092096 (PMC10180909; doi:10.3390/nu15092096)
Supplement: Supplementary file 1 [file nutrients-15-02096-s001.zip › nutrients-2328264-supplementary.pdf]

Table S1. Items of nutrition and health knowledge

Items of nutrition and health knowledge	Score ranges
**Core recommendations of dietary guidelines**	0-54
Q1. Recommendations on vegetable intake in the Dietary Guidelines for Chinese Residents (2016)	0-1.5
Q2. Recommendations on fruit intake in the Dietary Guidelines for Chinese Residents (2016)	0-1.5
Q3. Recommendations on dairy products intake in the Dietary Guidelines for Chinese Residents (2016)	0-1.5
Q4. Recommendations on soybean and its products intake in the Dietary Guidelines for Chinese Residents (2016)	0-1.5
Q5. Recommendations on meat intake in the Dietary Guidelines for Chinese Residents (2016)	0-1.5
Q6. Recommendations on egg intake in the Dietary Guidelines for Chinese Residents (2016)	0-1.5
Q7. Recommendations on processed meat intake in the Dietary Guidelines for Chinese Residents (2016)	0-1.5
Q8. Recommendations on sweet foods or beverage intake in the Dietary Guidelines for Chinese Residents (2016)	0-1.5
Q9. How much salt is recommended for healthy adults every day?	0-2
Q10. How much added sugar is recommended for healthy adults every day?	0-2
Q11. How much cooking oil is recommended for healthy adults every day?	0-2
Q12. Which of the following is more nutritious for lunch?	0-2
Q13. If an adult's body mass index is 26.1 kg/m^2, what is his/her weight classification?	0-2
Q14. Which of the following statements about vegetables and fruits are true? *	0-6
Q15. Which of the following statements about Dietary Guidelines for Chinese Residents are true? *	0-6
Q16. Which of the following can help maintain a healthy weight? *	0-6
Q17. Which of the following are the correct explanations for saving food? *	0-6
Q18. Which of the following are the correct explanations for dietary hygiene? *	0-8

**Food and nutrients**	0-19.5
Q19. Which food is best for supplementing calcium?	0-1.5
Q20. Compared with refined staple foods, what are the nutritional values of coarse cereals? *	0-6
Q21. Which of the following foods is rich in iron and is easily absorbed by the body? *	0-6
Q22. Which foods below can supplement vitamin A? *	0-6
**Nutrition and disease prevention**	0-15.5
Q23. Which food contains more cooking oil and salt?	0-1.5
Q24. Which food is most beneficial to prevent dyslipidemia and cardiovascular disease?	0-2
Q25. Which of the following statements about salt/sugared beverages and chronic disease are true? *	0-6
Q26. Which of the following statements about foods and chronic disease are true? *	0-6
**Nutrition skills**	0-11
Q27. Read the food labels below. Which product contains more protein?	0-1.5
Q28. Read the food labels below. Which product belong to dairy products?	0-1.5
Q29. How much does a handful of vegetables weigh？	0-2
Q30. How much does a palm-sized piece of lean meat weigh？	0-2
Q31. How much does an ordinary egg weigh?	0-2
Q32. How much does a fist-sized steamed bread weigh?	0-2
Total scores	0-100

* Multiple choice questions with 5 options.

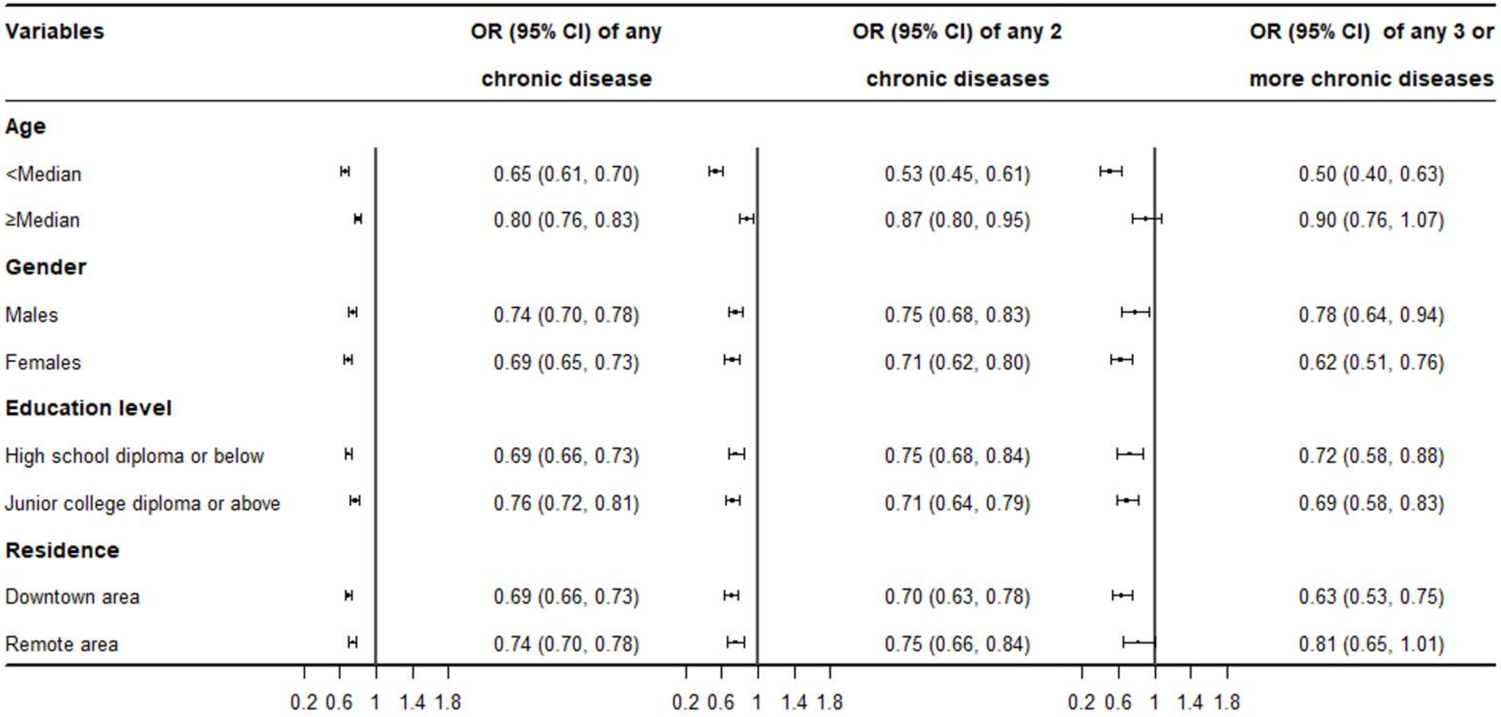

Figure S1. Stratified analysis of the association between per SD increment of NHK and the number of chronic diseases.
Adjusted for age, gender, education level, occupation, residence, knowledge acquisition from an app, and educational activities. The tests for interactions between stratified variables and NHK score were used likelihood ratio tests.
